# MiR-15b-5p and PCSK9 inhibition reduces lipopolysaccharide-induced endothelial dysfunction by targeting SIRT4

**DOI:** 10.1186/s11658-023-00482-5

**Published:** 2023-08-16

**Authors:** Elisa Martino, Nunzia D’Onofrio, Anna Balestrieri, Luigi Mele, Celestino Sardu, Raffaele Marfella, Giuseppe Campanile, Maria Luisa Balestrieri

**Affiliations:** 1 Department of Precision Medicine, University of Campania Luigi Vanvitelli, Via L. De Crecchio 7, 80138 Naples, Italy; 2 Food Safety Department, Istituto Zooprofilattico Sperimentale del Mezzogiorno, Via Salute 2, 80055 Portici, Italy; 3 Department of Experimental Medicine, University of Campania Luigi Vanvitelli, Via Luciano Armanni 5, 80138 Naples, Italy; 4 Department of Advanced Clinical and Surgical Sciences, University of Campania Luigi Vanvitelli, Piazza Miraglia, 80138 Naples, Italy; 5 Department of Veterinary Medicine and Animal Production, University of Naples Federico II, Via F. Delpino 1, 80137 Naples, Italy

**Keywords:** microRNA-15b-5p, PCSK9, SIRT4, Sepsis, Endothelial inflammation, Pyroptosis, Autophagy

## Abstract

**Background:**

Endothelial dysfunction and deregulated microRNAs (miRNAs) participate in the development of sepsis and are associated with septic organ failure and death. Here, we explored the role of miR-15b-5p on inflammatory pathways in lipopolysaccharide (LPS)-treated human endothelial cells, HUVEC and TeloHAEC.

**Methods:**

The miR-15b-5p levels were evaluated in LPS-stimulated HUVEC and TeloHAEC cells by quantitative real-time PCR (qRT–PCR). Functional experiments using cell counting kit-8 (CCK-8), transfection with antagomir, and enzyme-linked immunosorbent assays (ELISA) were conducted, along with investigation of pyroptosis, apoptosis, autophagy, and mitochondrial reactive oxygen species (ROS) by cytofluorometric analysis and verified by fluorescence microscopy. Sirtuin 4 (SIRT4) levels were detected by ELISA and immunoblotting, while proprotein convertase subtilisin-kexin type 9 (PCSK9) expression was determined by flow cytometry (FACS) and immunofluorescence analyses. Dual-luciferase reporter evaluation was performed to confirm the miR-15b-5p–SIRT4 interaction.

**Results:**

The results showed a correlation among miR-15b-5p, PCSK9, and SIRT4 levels in septic HUVEC and TeloHAEC. Inhibition of miR-15b-5p upregulated SIRT4 content, alleviated sepsis-related inflammatory pathways, attenuated mitochondrial stress, and prevented apoptosis, pyroptosis, and autophagic mechanisms. Finally, a PCSK9 inhibitor (i-PCSK9) was used to analyze the involvement of PCSK9 in septic endothelial injury. i-PCSK9 treatment increased SIRT4 protein levels, opposed the septic inflammatory cascade leading to pyroptosis and autophagy, and strengthened the protective role of miR-15b-5p inhibition. Increased luciferase signal validated the miR-15b-5p–SIRT4 binding.

**Conclusions:**

Our in vitro findings suggested the miR-15b-5p–SIRT4 axis as a suitable target for LPS-induced inflammatory pathways occurring in sepsis, and provide additional knowledge on the beneficial effect of i-PCSK9 in preventing vascular damage by targeting SIRT4.

**Graphical Abstract:**

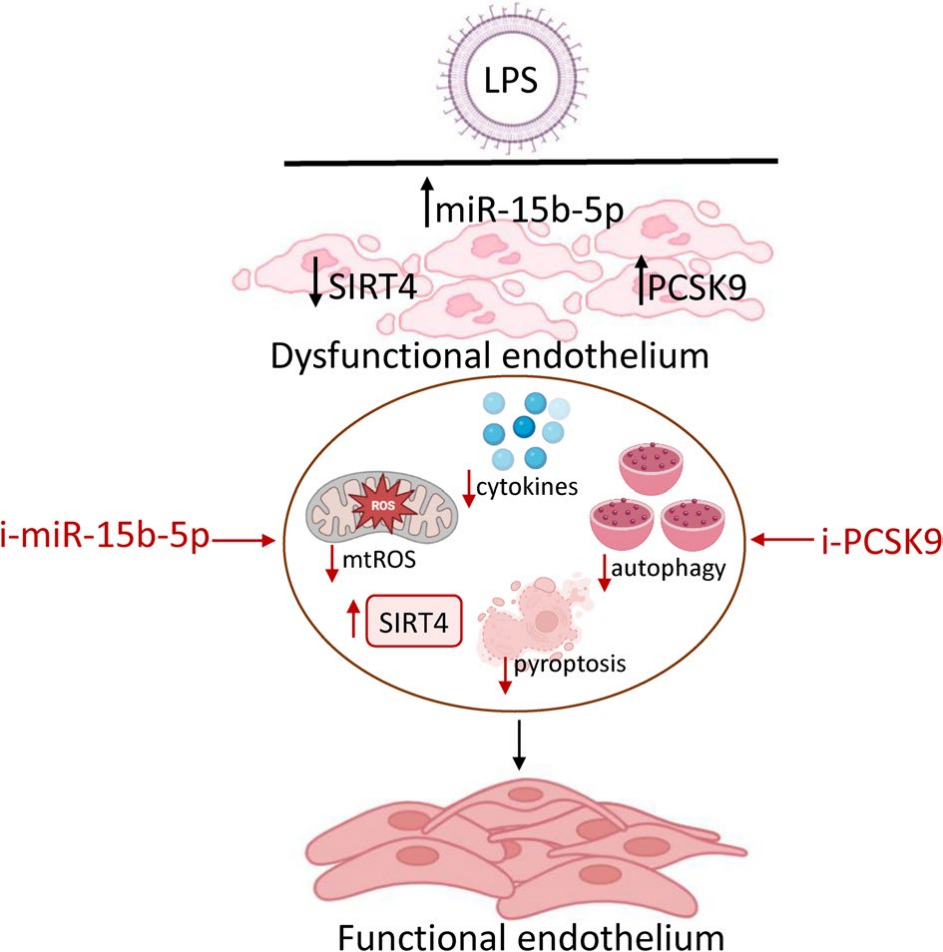

**Supplementary Information:**

The online version contains supplementary material available at 10.1186/s11658-023-00482-5.

## Background

Endothelial dysfunction, a critical hallmark of vascular homeostasis deregulation and cardiovascular disease pathogenesis [1, 2], is related to sepsis, a severe condition leading to irreversible acute inflammatory response, multiple organ dysfunction, poor prognoses, susceptibility to secondary infections, and mortality [2–4]. Although current evidence revealed that endothelium protection is functional in sepsis treatment [5], the detailed mechanisms of septic endothelial dysfunction are not fully understood. The endotoxin or lipopolysaccharide (LPS) is a potent proinflammatory element associated to endothelial cell (EC) injury and apoptosis [5, 6]. LPS elicits multiple endothelial responses, including the release of tissue factor, cytokines, and adhesion molecules, as vascular cell adhesion molecule-1 (VCAM-1), intercellular adhesion molecule 1 (ICAM-1), interleukin-1β (IL-1β), IL-18, and monocyte chemoattractant protein-1 (MCP-1) [7]. In EC, LPS increases mitochondrial reactive oxygen species (ROS) as well as Lectin-like ox-LDL receptor-1 (LOX-1) and proprotein convertase subtilisin/kexin type 9 (PCSK9) expression [8], belonging to the proprotein convertase family, regulating low-density lipoprotein (LDL) and cholesterol metabolism [9]. PCSK9, the attractive molecular target for controlling and decreasing the risk of cardiovascular diseases [10], is involved in the evolution of atherosclerotic plaques, while the PCSK9 inhibitor (i-PCSK9), evolocumab, displays beneficial effects in vascular function [11]. To date, the action of PSCK9 in sepsis has not been established [12]. Indeed, PCSK9 has been reported as a valuable therapeutic target for sepsis [13]. Furthermore, its loss in LPS-exposed EC exacerbates the proinflammatory response and increases LPS uptake, thus indicating that PCSK9 repression may counteract the therapeutic action of PCSK9 inhibition [14].

Evidence on the pathogenesis of endothelial dysfunction and sepsis revealed the prognostic and diagnostic potentials of microRNAs (miRNAs) [15–19], short noncoding single-stranded RNAs, with posttranscriptive gene silencing features arising by interaction with the 3′-untranslated region (3′-UTR) of target mRNAs [20, 21]. EC dysfunction and miRNAs represent key targets for the septic inflammatory process. Therefore, it is reasonable that miRNA regulation could represent a useful therapeutic tool for sepsis-induced EC damage.

MiR-15b, belonging to the conserved miR-15/16 family, is highly expressed in EC and displays multiple roles in different cell and tissue environments by regulating endothelium functions [22, 23]. This miR family includes miR-15a/16-1, miR-15b/16-2, and miR-497/195, sharing conserved sequences and target genes (https://www.targetscan.org/cgi-bin/targetscan/vert_80/targetscan.cgi?species=Human&gid=&mir_sc=&mir_c=&mir_nc=&mir_vnc=&mirg=hsa-miR-15b-5p) and allowing that the loss of one cluster can be functionally counterbalanced by other family members. In the context of endothelial biology, miR-15b regulates proliferation, invasion, and angiogenesis [24]. The overexpression of miR-15b in human umbilical vein endothelial cells (HUVEC) inhibited angiogenesis, while the attenuation of endogenous miR-15b expression improved vascular endothelial growth factor receptor 2 (VEGFR2) levels and HUVEC migration [23]. Moreover, miR-15b prevents vascular endothelial growth factor (VEGF) and angiopoietin-2 (Ang-2) expression, suggesting it as an anti-angiogenesis target [25]. Additionally, miR-15b/16 overexpression downregulates tumor necrosis factor-α (TNF-α) and suppressor of cytokine signaling 3 (SOCS3) protein levels and enhances insulin-like growth factor binding protein-3 (IGFBP-3) expression, thus protecting retinal EC from apoptosis related to hyperglycemic stress [26].

Among the miR-15 family, miR-16 has been shown as vital player in sepsis [27–29]. Deprivation of miR-16 notably reduces the mortality rates in different septic models [30, 31], showing a positive correlation between miR-16 levels in serum and death in septic patients [32]. It has been reported that miR-195 is able to promote apoptotic mechanism by targeting the SIRT1/eukaryotic translation initiation factor 2A (eIF2a) in intestinal epithelial cells infected with LPS, and increased miR-195-5p levels were also assessed in both human small airway epithelial cells exposed to LPS and in lung tissues from septic rats [33, 34]. MiR-15a-5p antagomiR denies the inflammatory pathway in both macrophages and septic mouse models via nuclear factor kappa B (NF-κB) signaling inhibition and targeting TNF-α induced protein 3 (NFAIP3)-interacting protein 2 (TNIP2) [35]. MiR-15a and miR-16 expression are improved in the blood of septic newborn subjects and inhibit the LPS-promoted inflammation [36], and miR-15b drives the onset and evolution of sepsis in animal models [37]. Research shows that miR-15b-5p expression predicts COVID-19 severity and it is involved in mammalian target of rapamycin (mTOR) signaling pathway in sepsis-induced acute kidney injury [37, 38]. However, the mechanistic role of miR-15b in septic EC has not yet been clearly explained.

Emerging evidence supports the role of sirtuins (SIRT1-7) in the progression and prognosis of sepsis acting on epigenetic profile [39–42], even if the involvement of SIRT4 in endothelial septic disorder is widely debated. Of note, SIRT4 emerges as a physiological player leading to hypo-inflammation and promoting sepsis recovery [43]. SIRT4-silenced EC showed an exacerbated inflammatory stress, while its upregulation reduces LPS-related inflammation [44]. Therefore, SIRT4 might represent a promising candidate to counteract the in vitro endotoxin-mediated cytotoxicity.

Here, we provide the in vitro miR-15b-5p implication in the EC inflammatory response during sepsis and the possible relationship with SIRT4. In detail, we investigated the molecular mechanism(s) of EC dysfunction occurring under LPS exposure and the ability of miR-15b-5p inhibition (i-miR-15b) to oppose the LPS-derived pro-inflammatory and pro-autophagic phenomena by modulating SIRT4 and PCSK9 levels. In this context, an expanding body of research has revealed the strong interplay between PCSK9 and miRNA [45–49]. Therefore, given that an interesting relationship also exists between i-PCSK9 and endothelial function [11], we examined its ability in LPS-induced EC inflammation, thus contributing to the design of new approaches for sepsis treatment.

## Methods

### Endothelial cell growth and stimulation

EC from human aorta (TeloHAEC, CRL-4052, ATCC, Manassas, VA, USA) and primary human umbilical vein (HUVEC, PCS-100-010, ATCC, Manassas, VA, USA) were grown as previously reported [50]. To mimic septic inflammatory condition, EC were treated up to 24 h with increasing concentrations (0.5–10 µg/mL) of lipopolysaccharides (LPS, L5543, Sigma-Aldrich, St. Louis, MO, USA). To selectively inhibit proprotein convertase subtilisin/kexin type 9 (PCSK9), EC were incubated with 100 µg/mL of evolocumab (i-PCSK9, Repatha, Amgen Europe B.V) along with LPS 10 µg/mL for 24 h. TeloHAEC were transfected with 2 µg antagomiR hsa-miR-15b-5p (i-miR-15b, customized by Aurogene, Rome, Italy) or with corresponding antagomir Negative Control (NC, Aurogene, Rome, Italy) using the vehicle Lullaby (LL70500, OZ Biosciences, Marseille, France) in medium without serum and antibiotic. After 6 h, fetal bovine serum was added to the culture medium and incubated for further 12 h before treatments with LPS and/or i-PCSK9. The miR-15b-5p inhibition efficiency after transfection was assessed by quantitative real-time PCR (qRT–PCR). Control cells (Ctr) were grown as previously reported [11].

### Viability and cytotoxicity detection

Cell viability and cytotoxicity assays were assessed as previously described [11, 50], following the instructions of the manufacturer. The lactate dehydrogenase (LDH) release (%) in culture media was used to determine cytotoxicity. Data are from *n* = 3 independent experiments.

### Nitric oxide (NO) levels

The amount of NO was assessed by Nitric Oxide assay kit, as previously described [50]. The NO levels were estimated by interpolating the sample OD with the standard curve and reported as µM.

### ELISA assays

Inflammatory mediators (VCAM1, ab223591; ICAM1, ab174445; MCP-1, ab179886; IL-1β, ab214025; IL-18, ab215539, all from Abcam, Cambridge, UK), caspase-4 (ab275098, Abcam, Cambridge, UK), PCSK9 (DPC900, R&D Systems, Minneapolis, MN, USA, Inc.), SIRT4 (MBS2705670, MyBioSource, San Diego, CA, USA), and LOX-1 (EHOLR1, Invitrogen, Waltham, MA, USA) levels were determined by human enzyme-linked immunosorbent assays (ELISA), following the specific instructions.

The 450 nm absorbance was detected with a reader (Bio-Rad, Hercules, CA, USA) and levels of cytokines, caspase-4, SIRT4, PCSK9, and LOX-1 in the samples were calculated using standard curve.

### Caspase-4 activity

Caspase 4 activity was determined by a fluorometric kit (ab65658, Abcam, Cambridge, UK), according to the protocol. Fluorescence (ex. 400 nm/em. 505 nm) was detected with a reader (Tecan, Männedorf, Switzerland).

### Quantitative real-time PCR

The evaluation of hsa-miR-15b-5p, hsa-miR-16-5p, hsa-miR-195-5p, and PCSK9 mRNA levels was performed as previously reported [50]. ID3EAL Individual miRNA RT Primer 1-plex (103113-HSA0000417A, 1103113-HSA0000069A, and 1103113-HSA0000461A, all from MiRXES, Singapore) and ID3EAL miRNA qPCR (1104101-HSA0000417A, 1103114-HSA0000069A, and 1104101-HSA0000461A, all from MiRXES, Singapore) primers were used to quantify hsa-miR-15b-5p, hsa-miR-16-5p, and hsa-miR-195-5p levels, respectively, while the assessment of PCSK9 mRNA was carried with the following primer:

PCSK9 (255738): F-AGGGGAGGACATCATTGGTG, R-CAGGTTGGGGGTCAGTACC and normalized against GAPDH [50]. The relative amount was determined using the 2^−ΔΔCt^ method [50].

### Cell death detection

The different mechanisms of cell death as pyroptosis, lysosome accumulation, autophagy, and apoptosis were evaluated by fluorescent image acquisition and flow cytometry analyses, as previously described [11, 50, 51].

### Mitochondrial ROS detection

Mitochondrial ROS accumulation was measured using MitoSOX Red probe, as previously reported [11, 50].

### Western blotting

Protein lysis, sodium dodecyl sulfate–polyacrylamide gel electrophoresis (SDS–PAGE), nitrocellulose membrane transfer, and chemiluminescent acquisition and analysis were conducted as already described [11, 50] with anti-microtubule-associated proteins 1A/1B light chain 3B (LC3B II/I, 1:2000, ab192890, Abcam, Cambridge, UK), anti-PCSK9 (1:1000, ab185194, Abcam, Cambridge, UK), anti-SIRT4 (1:1000, ab231137, Abcam, Cambridge, UK), anti-α-tubulin (1:5000, E-AB-20036, Elabscience Biotechnology Inc., Houston, TX, USA), and anti-actin (1:3000, ab179467, Abcam, Cambridge, UK) as primary antibodies.

### NLRP3 and PCSK9 intracellular detection by flow cytometry

EC were detached by trypsinization, and the Cytofix/Cytoperm Fixation/Permeabilization kit (554714, BD Biosciences, San Diego, CA, USA) was used following the protocol to fix and permeabilize. Thereafter, primary antibodies anti-NLR family pyrin domain-containing 3 (NLRP3, 10 µg/ml, ab4207, Abcam, Cambridge, UK) and -PCSK9 (1:500) were incubated for 45 min, followed by 30 min incubation with specific 488-conjugated secondary antibodies (1:250). Flow cytometry analysis was performed as previously reported [11, 50].

### Confocal laser scanning microscopy

Immunofluorescence detection of PCSK9 was assessed in EC as previously reported [52]. Images were obtained with a LSM 700 confocal microscope while the analysis was estimated with ImageJ 1.52n software.

### Bioinformatics

Potential miR-15b-5p targets and a putative complementary site targeting the 3′-UTR of SIRT4 mRNA were estimated using the miRDB (https://mirdb.org/cgi-bin/search.cgi), mirDIP (https://ophid.utoronto.ca/mirDIP/index.jsp#r), and TargetScan (https://www.targetscan.org/cgi-bin/targetscan/vert_80/targetscan.cgi?species=Human&gid=&mir_sc=&mir_c=&mir_nc=&mir_vnc=&mirg=hsa-miR-15b-5p) databases.

### Luciferase assay

The dual-luciferase vector was customized by Tetubio (Milan, Italy) using the pEZX–MT06 vector (GeneCopoeia, Rockville, MD, USA) containing the full SIRT4 3′-UTR binding sequence (217HmiT128732-MT-06). The dual-luciferase reporter analysis was conducted following a previous approach [50].

### Statistical analysis

Statistical analyses were carried by GraphPad Prism software (La Jolla, CA, USA), version 9.1.2, and the results are shown as the mean ± standard deviation (SD) of three independent experiments. To determine statistically significant differences, Student’s *t*-tests were used between two groups, and one-way ANOVAs for more than two groups. Differences with *p* < 0.05 were evaluated as statistically significant.

## Results

### LPS-induced inflammatory state

**Fig. 1:**
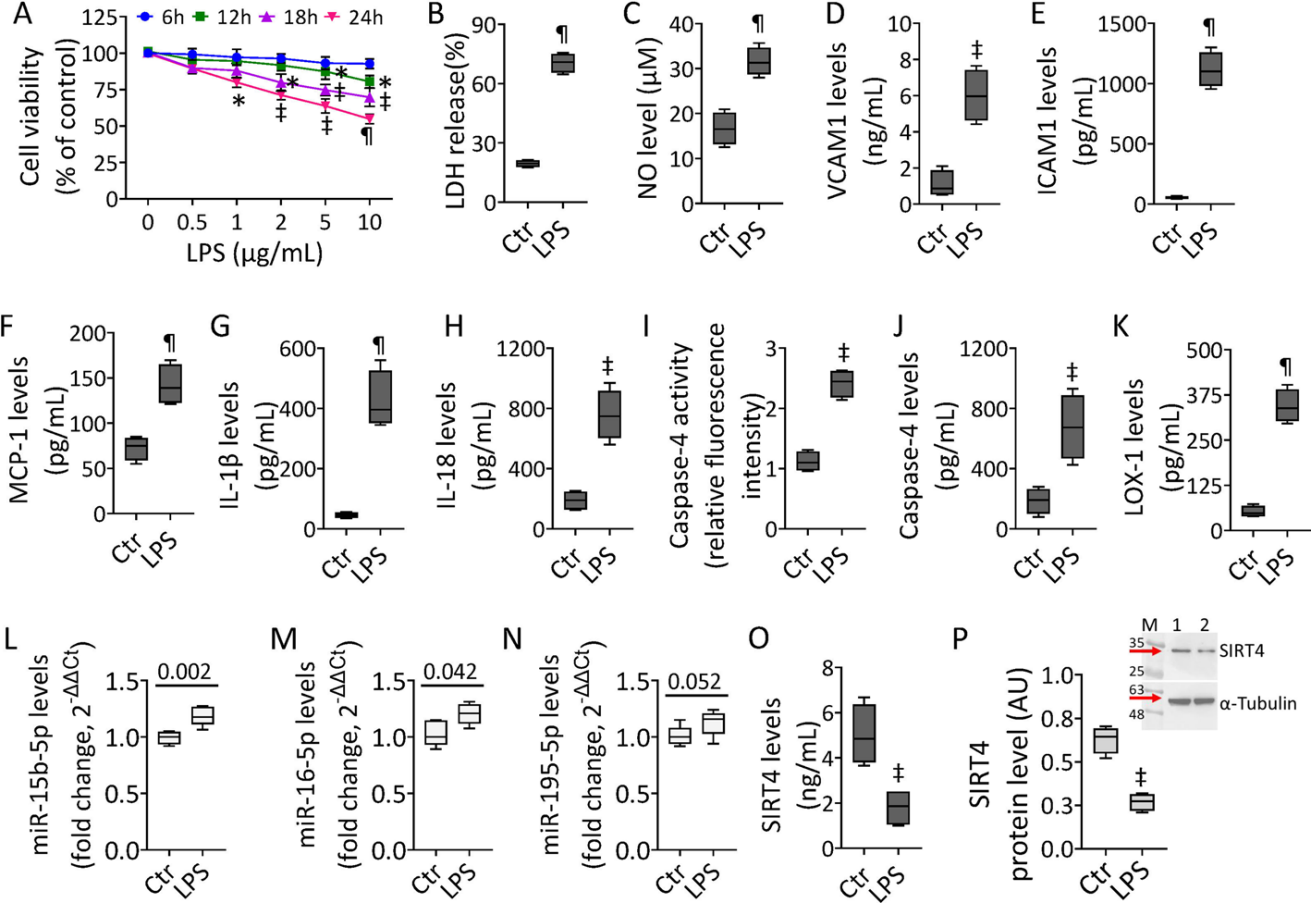
LPS-mediated inflammation. **A** TeloHAEC viability following LPS exposure. Detection of **B** LDH, **C** NO, **D** VCAM1, **E** ICAM1, **F** MCP-1, **G** IL-1β, and **H** IL-18 levels. Evaluation of caspase-4 **I** activity and **J** levels and **K** LOX-1 content. Representation of **L** hsa-miR-15b-5p, **M** hsa-miR-16-5p, and **N** hsa-miR-195-5p levels. SIRT4 levels assessed by **O** ELISA assay and **P** immunoblotting. M, molecular weight markers; lane 1, Ctr; lane 2, LPS. Mean ± SD, *n* = 3. *p < 0.05 versus 0 µg/mL or Ctr, ^‡^*p* < 0.01 versus 0 µg/mL or Ctr, ^¶^*p* < 0.001 versus 0 µg/mL or Ctr. Statistical analysis of data was performed using Student’s *t*-tests

To determine the molecular and functional activities of the proangiogenic miR-15b-5p in endothelial dysfunction occurring under septic stress, experiments were performed on teloHAEC and HUVEC cells treated with LPS. Dose–response experiments showed that LPS treatment exerted cytotoxicity in EC after 24 h incubation with 10 µg/mL (*p* < 0.001) (Fig. 1 and Additional file 1: Fig. S1). Exposure to LPS increased LDH release, upregulated NO levels, and triggered the inflammatory cascade, as evidenced by VCAM1, ICAM1, MCP-1, IL-1β, and IL-18 overexpression (*p* < 0.001) (Fig. 1 and Additional file 1: Fig. S1). Endotoxin promoted caspase-4 activation (*p* < 0.01) and LOX-1 accumulation (*p* < 0.001) (Fig. 1 and Additional file 1: Fig. S1). The expression of miR-15/16 family members, critical in LPS-mediated dysfunction, as miR-15b-5p, miR-16-5p, and miR-195-5p [27–38] was then assessed. The results show the ability of endotoxin treatment to upregulate miR-15b-5p, miR-16-5p, and miR-195-5p, with miR-15b-5p displaying the most significant increase in both teloHAEC and HUVEC cells (Fig. 1 and Additional file 1: Fig. S1). In addition, immunoblotting and ELISA experiments indicated the LPS-induced downregulation of mitochondrial SIRT4 levels (*p* < 0.01) (Fig. 1 and Additional file 1: Fig. S1).

### LPS effects on PCSK9 levels

**Fig. 2:**
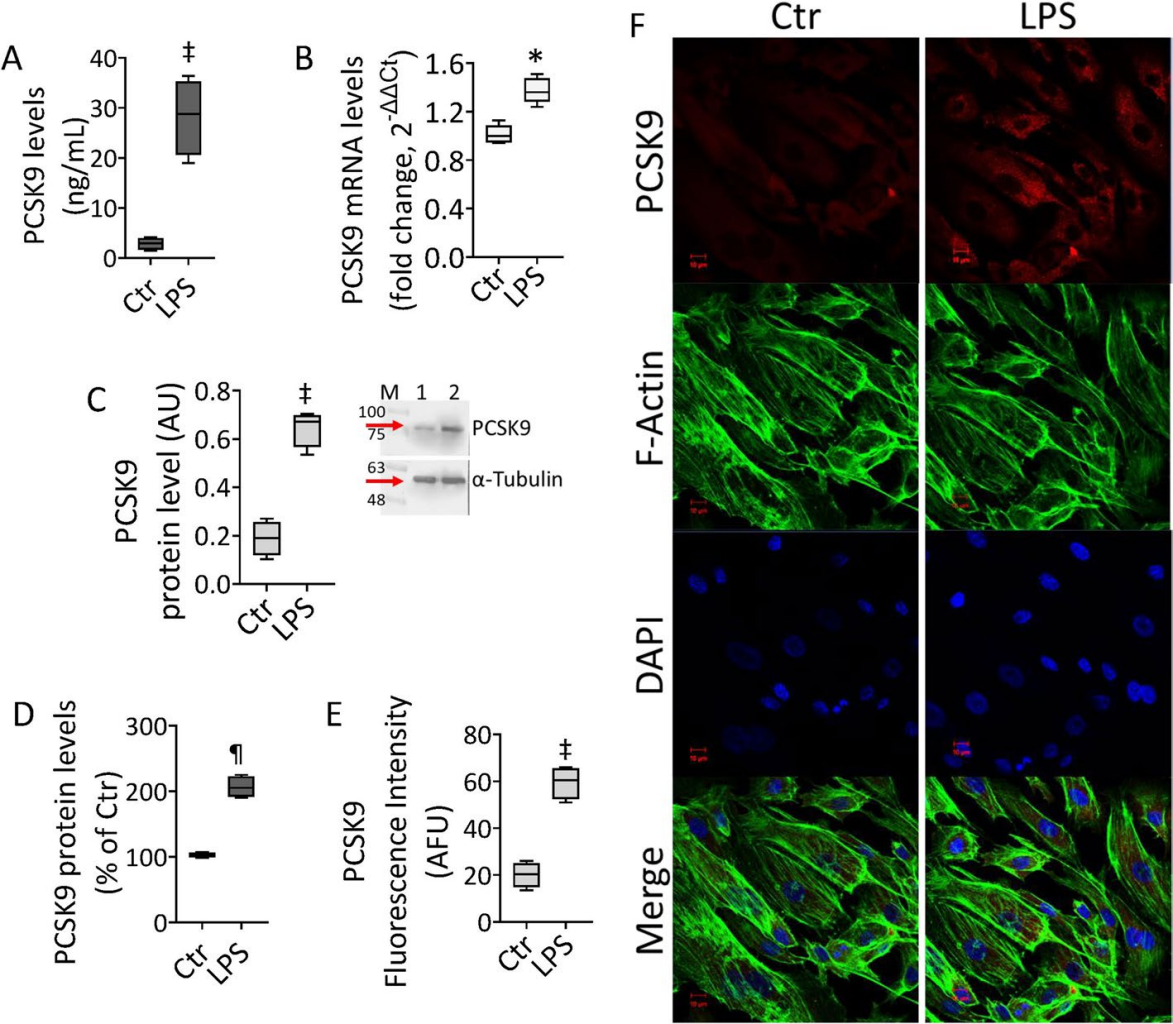
LPS modulation of PSCK9 protein. PCSK9 levels assessed by **A** ELISA assay, **B** mRNA by qRT–PCR, **C** immunoblotting analysis, **D** intracellular content by FACS analysis, and **E**, **F** confocal laser scanning microscopy. Mean ± SD, *n* = 3. M, molecular weight markers; lane 1, Ctr; lane 2, LPS. Scale bars = 10 μm. *p < 0.05 versus Ctr, ^‡^*p* < 0.01 versus Ctr, ^¶^*p* < 0.001 versus Ctr. Statistical analysis of data was performed using Student’s *t*-tests

Given the crucial role of PCSK9 in EC dysfunction and vascular diseases, the effects of LPS on PCSK9 expression have been investigated. Treatment with endotoxin led to PCSK9 accumulation in EC lines, as assessed by different experimental approaches (Fig. 2 and Additional file 1: Fig. S2). In detail, treated EC showed upregulated PCSK9 content in culture media (*p* < 0.01), mRNA (*p* < 0.05), and protein expression (*p* < 0.01) (Fig. 2 and Additional file 1: Fig. S2). The immunoblotting data were confirmed by intracellular PCSK9 detection through FACS evaluation and immunofluorescent analysis (*p* < 0.001) (Fig. 2D–F and Additional file 1: Fig. S2).

### LPS triggered cell death

**Fig. 3:**
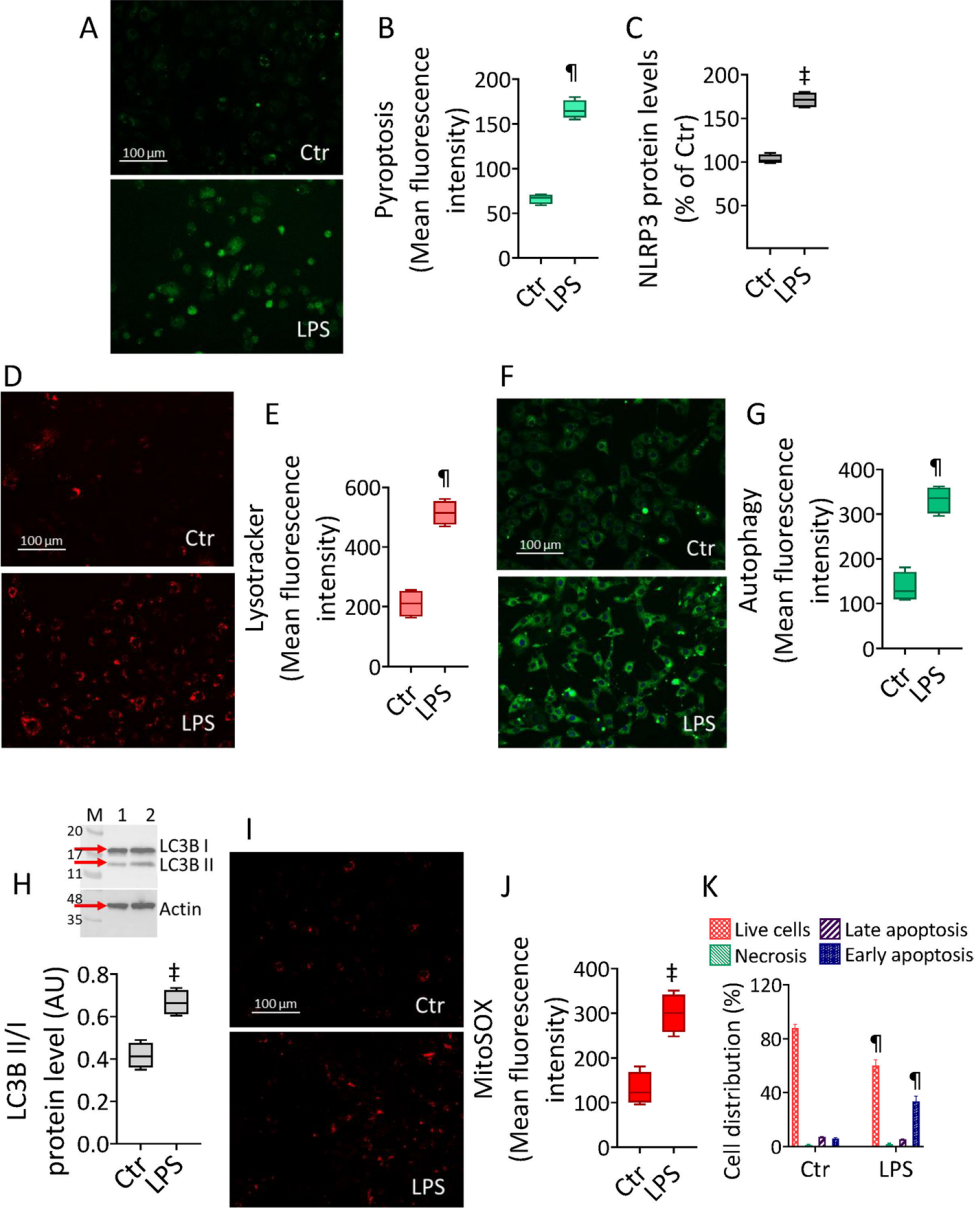
LPS outcomes on programmed cell mechanisms. **A** Representative images and **B** FACS detection of pyroptosis. **C** Intracellular NLRP3 levels assessed by FACS analysis. Representative images and cytofluorimetric evaluation of **D**, **E** lysosomes, **F**, **G** autophagy, and **H** immunoblotting of LC3B II/I. **I** Representative images and **J** FACS-detected mitochondrial ROS levels. **K** Representative annexin V-FITC and PI-staining detected by FACS analysis. Mean ± SD, *n* = 3. M, molecular weight markers; lane 1, Ctr; lane 2, LPS. Scale bars = 100 μm. ^‡^*p* < 0.01 versus Ctr, ^¶^*p* < 0.001 versus Ctr. Statistical analysis of data was performed using Student’s *t*-tests

The ascertained LPS-mediated inflammatory cascade prompted us to investigate the occurrence of programmed cell death mechanisms, as apoptosis and pyroptosis, and their association with the autophagic phenomenon. Exposure to LPS led to the induction of pyroptosis and inflammasome formation, as confirmed by increased NLRP3 protein levels (*p* < 0.01) (Fig. 3A–C and Additional file 1: Fig. S3). Treatment with endotoxin also induced lysosome accrual and autophagic flux, accompanied by extended pro-autophagic LC3B II/I ratio in TeloHAEC cells (*p* < 0.001) (Fig. 3D–H and Additional file 1: Fig. S3). The activation of these mechanisms was accompanied by LPS-promoted mitochondrial ROS production (*p* < 0.01) and apoptotic cell death (*p* < 0.001) (Fig. 3I–K and Additional file 1: Fig. S3).

### i-miR-15b-5p attenuated LPS-induced inflammation

**Fig. 4:**
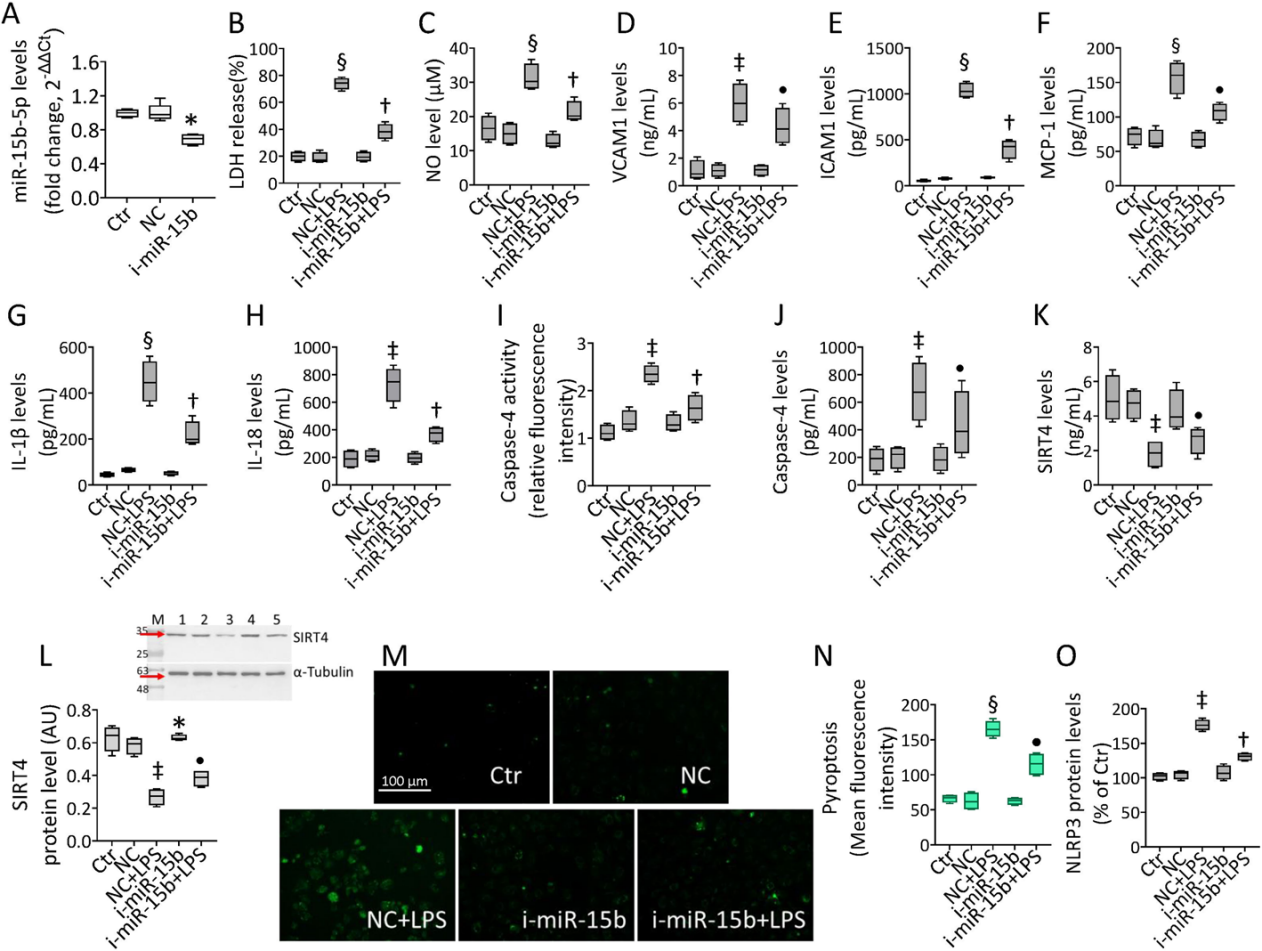
i-miR-15b opposed the LPS-related inflammation. **A** Hsa-miR-15b-5p expression measured by qRT–PCR in TeloHAEC transfected with antagomir negative control (NC) or antagomiR hsa-miR-15b-5p (i-miR-15b). Detection of **B** LDH, **C** NO, **D** VCAM1, **E** ICAM1, **F** MCP-1, **G** IL-1β, and **H** IL-18 levels. Assessment of caspase-4 **I** activity and **J** levels. SIRT4 levels assessed by **K** ELISA assay and **L** immunoblotting. **M** Representative images and **N** FACS analysis of pyroptosis. **O** Intracellular NLRP3 levels assessed by FACS detection. Mean ± SD, *n* = 3. M, molecular weight markers; lane 1, Ctr; lane 2, NC; lane 3, NC + LPS; lane 4, i-miR-15b; lane 5, i-miR-15b + LPS. Scale bars = 100 μm. *p < 0.05 versus NC, ^‡^*p* < 0.01 versus NC, ^§^*p* < 0.001 versus NC, ^•^*p* < 0.05 versus NC + LPS, ^†^*p* < 0.01 versus NC + LPS. Statistical analysis of data was performed using one-way ANOVAs.

Given the above concordant results between teloHAEC and HUVEC cells to LPS stimulation, further studies aimed to understand the molecular mechanism(s) of EC dysfunction under septic conditions were performed on teloHAEC. To test whether hsa-miR-15b-5p inhibition was able to prevent the inflammatory stress caused by LPS, teloHAEC were transfected for 18 h with hsa-miR-15b-5p antagomiR (i-miR-15b) before 24 h stimulation with LPS (Fig. 4A). Transfection with i-miR-15b up to 72 h displayed no cytotoxicity (Additional file 1: Fig. S4A). EC transfected with negative control antagomiR (NC) and exposed to increasing doses of LPS confirmed the cytotoxicity induction after 24 h incubation with 10 µg/mL (*p* < 0.001) (Additional file 1: Fig. S4B). Interestingly, i-miR-15b counteracted the LPS-induced LDH and NO release (*p* < 0.01 versus NC + LPS) (Fig. 4 B, C), as well as VCAM1, ICAM1, MCP-1, IL-1β, and IL-18 cytokine release (*p* < 0.01 versus NC + LPS) (Fig. 4D–H). MiR-15b inhibition decreased the caspase-4 protein levels and activity (*p* < 0.01 versus NC + LPS) (Fig. 4I, J), along with the LPS-derived pyroptosis and NLRP3 upregulation (*p* < 0.01 versus NC + LPS) (Fig. 4M–O and Additional file 1: Fig. S4C, D). On the contrary, transfection with antagomiR increased the SIRT4 protein content (*p* < 0.05 versus NC) and i-miR-15b + LPS treatment partly restored the mitochondrial SIRT4 content, downregulated by LPS (*p* < 0.05 versus NC + LPS) (Fig. 4K, L).

### i-miR-15b-5p opposed the LPS-related dysfunction

**Fig. 5:**
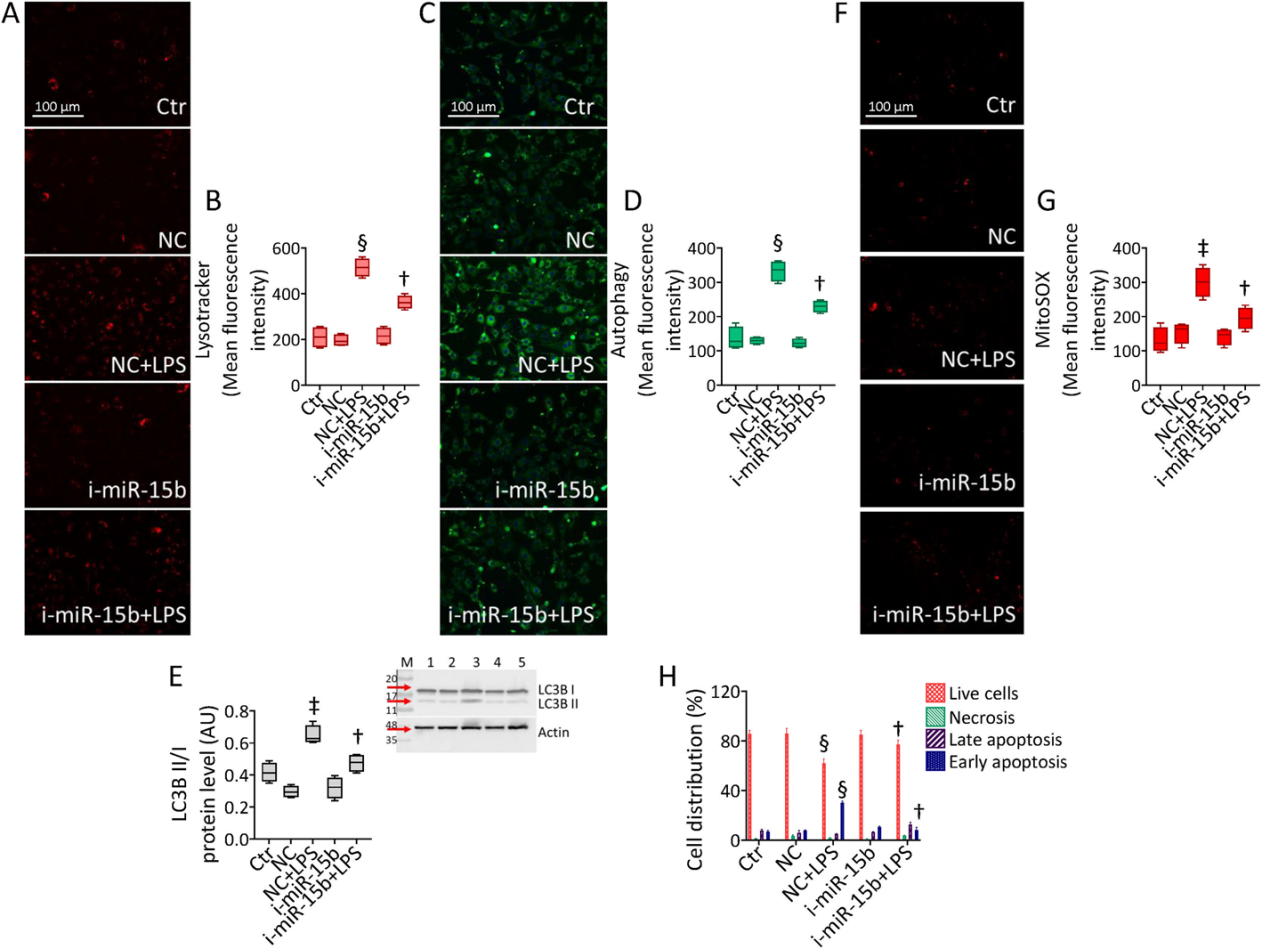
i-miR-15b counteracted the LPS-induced programmed mechanisms. Representative images and cytofluorimetric graph representation of **A**, **B** lysosomes, **C**, **D** autophagy, and **E** immunoblotting of LC3B II/I. **F**, **G** Representative images and graph of mitochondrial ROS and **H** annexin V-FITC and PI-staining detected by FACS analysis. Mean ± SD, *n* = 3. M, molecular weight markers; lane 1,  Ctr; lane 2, NC; lane 3, NC + LPS; lane 4, i-miR-15b; lane 5, i-miR-15b + LPS. Scale bars = 100 μm. ^‡^*p* < 0.01 versus NC, ^§^*p* < 0.001 versus NC, ^†^*p* < 0.01 versus NC + LPS. Statistical analysis of data was performed using one-way ANOVAs.

The protective activity exerted by i-miR-15b was evaluated against the main LPS-targeted endothelial dysfunction (Fig. 5 and Additional file 1: Fig. S5). The results revealed that the LPS-induced lysosome and autophagy induction was opposed by miR-15b inhibition (*p* < 0.01 versus NC + LPS) (Fig. 5A–D and Additional file 1: Fig. S5), also assessed by the restored protein ratio LC3B II/I (*p* < 0.01 versus NC + LPS) (Fig. 5E). Similarly, transfection with i-miR-15b counteracted the LPS-induced mitochondrial ROS accumulation and attenuated the apoptotic cell death occurrence (*p* < 0.01 versus NC + LPS) (Fig. 5F–H and Additional file 1: Fig. S5).

### i-PCSK9 and i-miR-15b-5p effects on LPS-induced inflammation

**Fig. 6:**
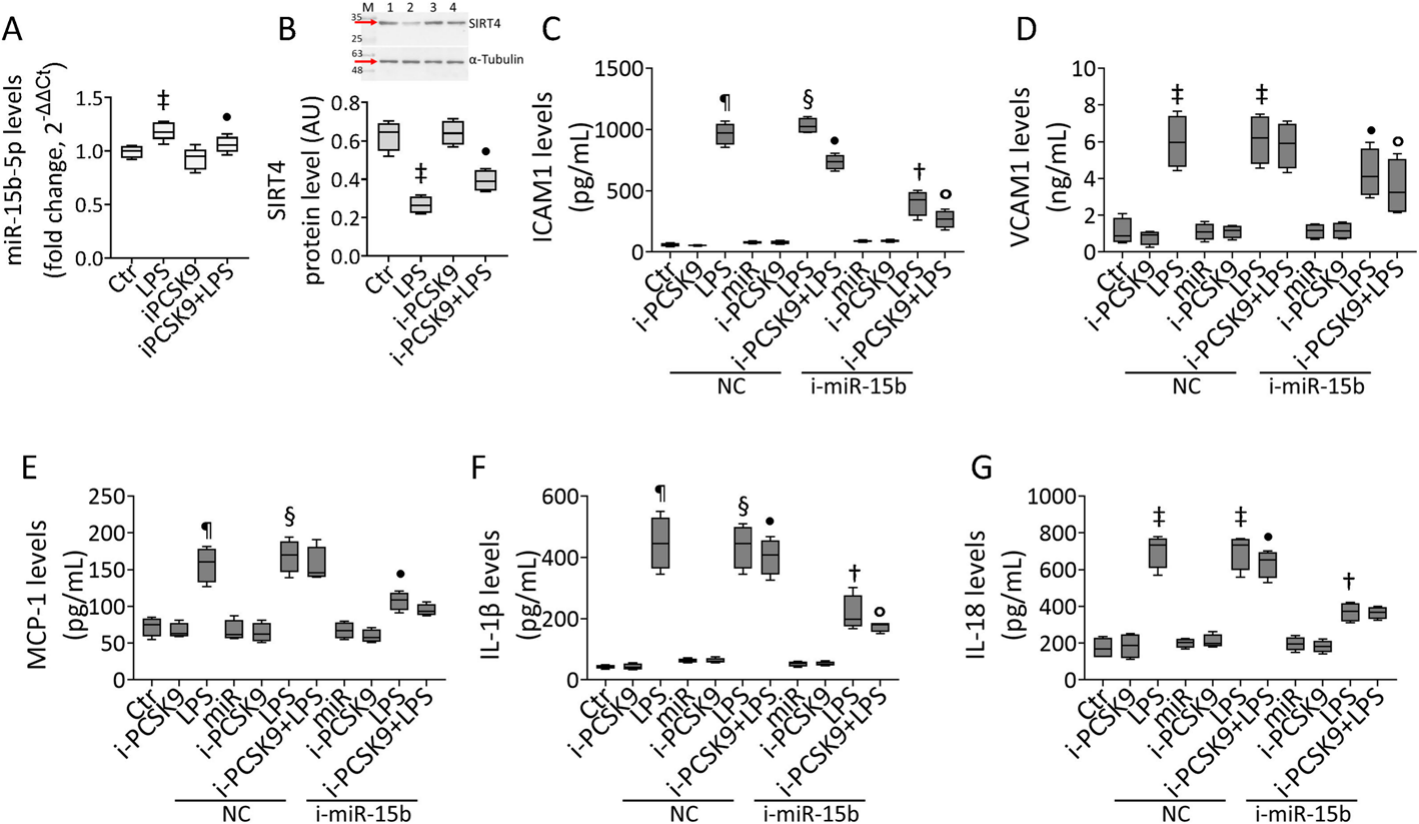
i-PCSK9 ameliorated the ability of i-miR-15b against LPS-related inflammation. **A** Hsa-miR-15b-5p levels and **B** immunoblotting of SIRT4. Mean ± SD, n = 3. M,  molecular weight markers; lane 1, Ctr; lane 2, LPS; lane 3, i-PCSK9; lane 4, i-PCSK9 + LPS. Detection of **C** ICAM1, **D** VCAM1, **E** MCP-1, **F** IL-1β, and **G** IL-18. Mean ± SD, *n* = 3. ^‡^*p* < 0.01 versus Ctr or NC, ^¶^*p* < 0.001 versus Ctr, ^§^*p* < 0.001 versus NC, ^•^*p* < 0.05 versus LPS or NC + LPS, ^†^*p* < 0.01 versus NC + LPS, °*p* < 0.05 versus i-miR-15b + LPS. Statistical analysis of data was performed using one-way ANOVAs.

Given that treatment with endotoxin led to PCSK9 accumulation in EC, the role of evolocumab, a PCSK9 inhibitor (i-PCSK9) employed in clinical practice, on the inflammatory pathways triggered by LPS was investigated (Fig. 6). The results showed that pretreatment with i-PCSK9 100 µg/mL for 8 h opposed the LPS-induced miR-15b-5p upregulation (*p* < 0.05) and SIRT4 decrease (*p* < 0.05) (Fig. 6A, B). Of interest, i-PCSK9 was also effective in inhibiting the LPS-related inflammatory cascade on transfected EC (Fig. 6). Indeed, ELISA showed the ability of i-PCSK9 to attenuate VCAM1, ICAM1, MCP-1, IL-1β, and IL-18 cytokine release (*p* < 0.05 versus NC + LPS) and to ameliorate the capacity of i-miR-15b to oppose endotoxin-induced inflammation (*p* < 0.05 versus i-miR-15b + LPS) (Fig. 6).

### i-miR-15b-5p–SIRT4 and i-PCSK9 counteracted LPS-related cytotoxicity

**Fig. 7:**
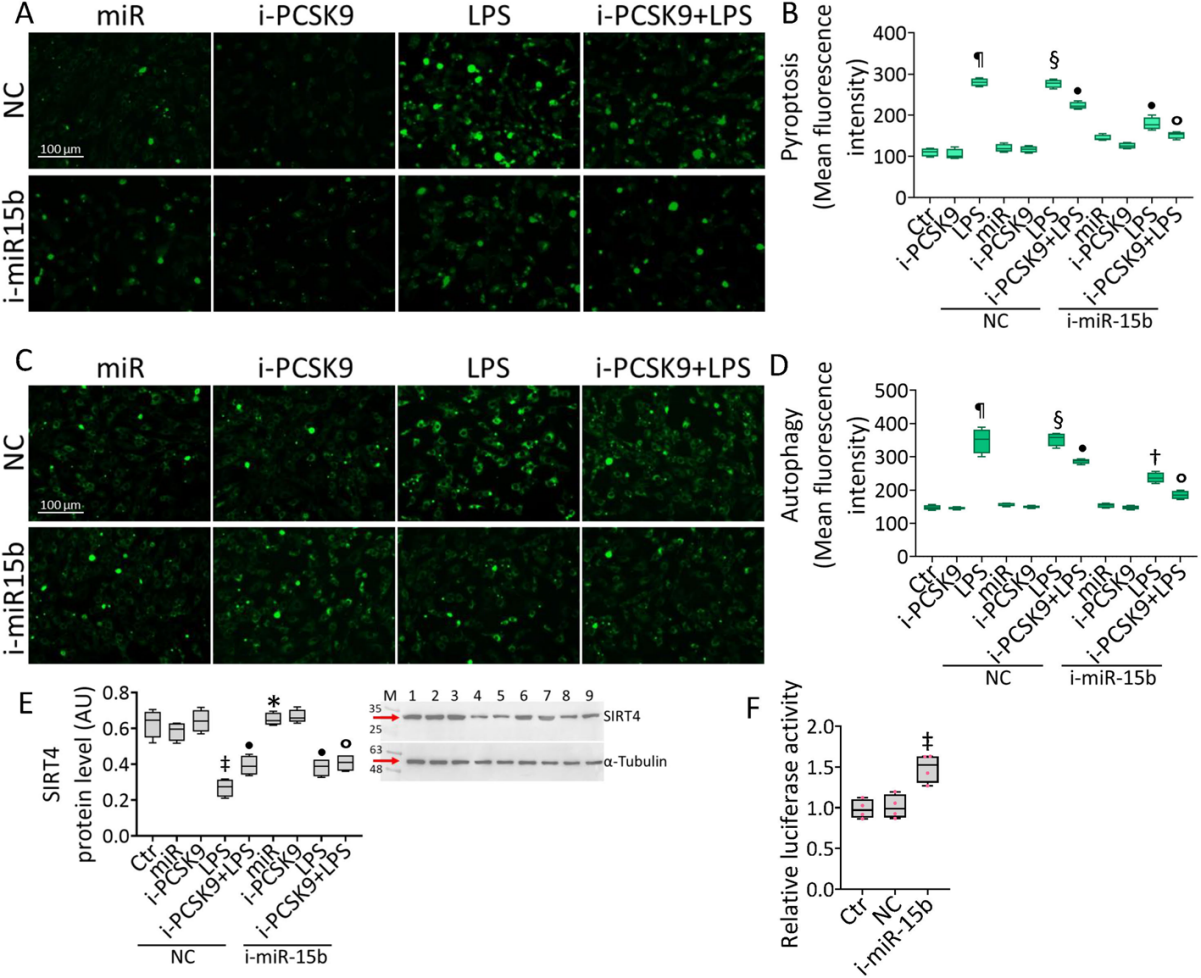
i-PCSK9 and i-miR-15b prevented LPS-related dysfunction. Representative images and graphical representation of FACS analysis of **A**, **B** pyroptosis and **C**, **D** autophagy. **E** Representative immunoblotting of SIRT4. Mean ± SD, *n* = 3. M, molecular weight markers; lane 1, Ctr; lane 2,  NC; lane 3,  NC + i-PCSK9; lane 4,  NC + LPS; lane 5,  NC + i-PCSK9 + LPS; lane 6, i-miR-15b; lane 7, i-miR-15b + i-PCSK9; lane 8, i-miR-15b + LPS; lane 9, i-miR-15b + i-PCSK9 + LPS. Scale bars = 100 μm. **F** The relative luciferase activity in EC co-transfected with luciferase reporter plasmid containing the SIRT4 3′-UTR sequence and with antagomir, NC or i-miR-15. *p < 0.05 versus NC, ^‡^*p* < 0.01 versus NC, ^¶^*p* < 0.001 versus Ctr, ^§^*p* < 0.001 versus NC, ^•^*p* < 0.05 versus NC + LPS, ^†^*p* < 0.01 versus NC + LPS, ^°^*p* < 0.05 versus i-miR-15b + LPS. Statistical analysis of data was performed using one-way ANOVAs.

The effects of i-PCSK9 on LPS-induced endothelial damage were then assessed (Fig. 7 and Additional file 1: Fig. S6). Treatment with i-PCSK9 opposed the induction of cytotoxicity, pyroptosis, and autophagy by LPS (*p* < 0.05 versus NC + LPS) (Fig. 7 and Additional file 1: Fig. S6) and improved the protective effects of i-miR-15b (*p* < 0.05 versus i-miR-15b + LPS) (Fig. 7 and Additional file 1: Fig. S6). Finally, i-PCSK9 enhanced the ability of i-miR-15b to restore the SIRT4 protein levels (*p* < 0.05 versus i-miR-15b + LPS) (Fig. 7E), indicating the pivotal role of the miR-15b-5p–SIRT4 axis in endothelial dysfunction under sepsis. To gain molecular insights about these findings, bioinformatic tools were used to reveal the SIRT4–mRNA 3ʹ-UTR region as a predictive binding site for hsa-miR-15b-5p. The 3′-UTR SIRT4 gene sequence was inserted downstream of the luciferase reporter vector and a dual-luciferase reporter analysis was carried on co-transfected (luciferase-i-miR-15b) EC (Fig. 7F). The results showed the ability of i-miR-15b to enhance the luciferase activity of 3′-UTR SIRT4 (*p* < 0.01), confirming the binding of hsa-miR-15b-5p with the 3′-UTR of SIRT4-mRNA (Fig. 7F).

## Discussion

The above results represent the first evidence of the role of miR-15b-5p in the endothelial impairment caused by sepsis. We found that during sepsis, the elevated miR-15b-5p levels were related to vascular endothelial cell damage, robust inflammation, activation of the NLRP3 pathway, increased PCSK9 levels, and downregulation of SIRT4. Our data suggest that miR-15b-5p, by targeting SIRT4, could represent a potential target in septic EC and advanced comprehension on the pleiotropic beneficial effects of i-PCSK9, able to ameliorate the endothelial inflammatory response during LPS treatment.

Sepsis is a complex pathophysiology with features of excessive inflammatory response leading to endothelial impairment [53]. Accordingly, our data show that the LPS displayed cytotoxicity in EC by increasing LDH content and NO levels, and promoting the massive release of inflammatory modulators as cytokines VCAM1, ICAM1, and MCP-1. The resulting systemic inflammation increases miR-15b-5p levels, as well as the expression of PCSK9, while a notable downregulation of SIRT4 protein levels was determined. In the sepsis-induced inflammatory mechanisms, evidence describes that miRNA deregulation might be related to clinical symptoms and disease severity.

Several miRNAs, including miR-93-5p, miR-16, miR-218-5p, miR-21-3p, miR-125b-5p, miR-133a, miR-146a, miR-297, and miR-122 have been detected as biomarkers in septic diagnosis [29, 32, 54–59]. Increased levels of miR-130b have been associated to suppression of lung inflammation due to LPS stimulation in a septic mouse model [60]. Moreover, miR-25 contributed to the treatment of sepsis, as well as miR-22-3p, which might be regarded as an early diagnostic biomarker for septic conditions [61]. MiR-15b-5p notably supports or opposes endothelial migration and proliferation, in both physiological conditions and altered environments as sepsis, by targeting the protein kinase B3 (AKT3) and ICAM1/ focal adhesion kinase (FAK) pathways, respectively [62, 63]. The promoting role of miR-15b-5p in hypoxia/reoxygenation and pyroptosis via NLRP3 inflammasome activation and SIRT3 modulation in cardiomyocytes has been described [64]. The NLRP3 inflammasome complex, consisting of NLRP3, apoptosis-associated speck-like protein containing CARD (ASC) and caspase-1, plays a fundamental role in dysfunctional endothelium during sepsis [65]. In human fibroblasts, cytoplasmic recognition of LPS is prompted by caspase-4 noncanonical inflammasomes, thus inducing inflammatory pyroptotic cell death and promoting senescence, associated to the caspase-4 substrate gasdermin-D and the tumor suppressor p53 [66]. Similarly, we showed that exposure to LPS increased caspase-4, both the level and activity, and triggered pyroptotic cell death sustained by NLRP3 activation. Our data indicate that activation of inflammasomes via caspase-4 and NLRP3 pathways, as well as the rise of inflammatory cytokines levels, were opposed by miR-15b-5p inhibition. Previous studies have shown that extensive inflammatory stress occurring under endothelial dysfunction is accompanied by lysosome and mitochondrial ROS accrual, as by autophagic flux [11, 51]. Consistently, we found that LPS promoted mitochondrial ROS accumulation and autophagy induction, whilst miR-15b-5p inhibition attenuated both phenomena.

Clinical studies have reported that patients with sepsis had increased sera levels of PCSK9, indicating PCSK9 as a biomarker of septic disorder [67], while other studies showed that PCSK9 content is positively associated with liver and kidney damage in septic mouse models [68]. Our study showed that PCSK9 is upregulated in EC during in vitro sepsis condition, while its pharmacological inhibition reduces vascular inflammation and improves endothelial function. Recent studies have described the ability of PCSK9 inhibitors, potent lipid-lowering agents, in decreasing the risk of sepsis [69–71]. Our previous data have shown that PCSK9 caused inflammatory stress and endothelial dysfunction, and that the pleiotropic protective effects of i-PCSK9 in EC might be mediated, at least in part, by NAD-dependent deacetylase SIRT3 [11]. Here, we substantiate previous data by showing that i-PCSK9 opposed the LPS-related inflammatory cascade, promoted SIRT4 upregulation, counteracted pyroptosis and autophagic mechanisms, and enhanced the positive action of miR-15b-5p repression on septic EC. Reports on the role of SIRT4 during sepsis are scarce. SIRT4 overexpression inhibits the mitochondrial metabolism, inflammation, and degranulation in LPS-stimulated mast cells, while the endotoxin triggers mitochondrial impairment via SIRT4 inhibition in LC-540 Leydig cells [72, 73]. Of note, both PCSK9 and miR-15b-5p repression exerted modulatory action on SIRT4 protein levels, counteracting the LPS-induced depletion, suggesting mitochondrial SIRT4 as a promising target in the septic phenotype. The functional effects mediated by miR-15b-5p can be attributed, at least partly, to its capacity to modulate SIRT4 expression. However, the role of SIRT4 in damaged endothelium and its involvement in septic disease require further investigations. The limitations of our study are due to the experiments based on in vitro cell line model. The use of animal models should be used to further understand the pathogenesis of sepsis. Among these models, cecal ligation and puncture in rodents may contribute to the definition of mechanistic roles of miR-15b in the regulation of EC pathophysiological responses associated with sepsis, as well as to clarify the expression modulation of sepsis-related miRNAs in EC [29, 74]. Following this experimental strategy, Wu et al. identified a panel of sepsis-induced miRNAs and their transcription regulators by array and immunoprecipitation analyses performed on blood samples from septic mice that had undergone cecal ligation and puncture [74]. The use of genomic and sequencing approaches could enhance comprehension on the intricate networks regulating the gene expression mediated by miR-15b in septic EC. Because miR-15b sharing the same cluster could emphasize its functional cooperation on LPS-mediated endothelial dysfunction, further investigations are needed to understand the contributing role of miR-15/16 family members in the development and progression of human septic conditions. In this scenario, it has to be taken into account the fundamental regulatory role of other noncoding RNAs, as long noncoding RNAs (lncRNAs), able to negatively modulate miRNA functions. Indeed, additional studies are undoubtedly necessary to expand the cell-based results with a mechanistic approach and translate this notion to human or animal diseases, which may improve knowledge to better understand and treat septic conditions.

## Conclusions

The present study provides in vitro evidence on PCSK9 and miR-15b-5p repression as potent antiinflammatory, antipyroptotic, and antiautophagic mechanisms by modulating the mitochondrial SIRT4 protein levels. In summary, miR-15b-5p inhibition opposed the NLRP3 pathways related to inflammatory stress and impaired endothelium during sepsis. Furthermore, suppression of PCSK9 may be a promising tool in septic therapy when miR-15b-5p is silenced. Overall, these results demonstrate that i-PCSK9 protected endothelium in sepsis via SIRT4, indicating this epigenetic modulator as a possible innovative target against endothelial dysfunction induced by sepsis.

### Supplementary Information

**Additional file 1:** **Figure S1.** LPS-mediated inflammation on HUVEC cells. **A** Cell viability on HUVEC exposed to LPS. Detection of **B** LDH, **C** NO, **D** VCAM1 **E** ICAM1, **F** MCP-1, **G** IL-1β and **H** IL-18 levels. Caspase-4 **I** activity and **J** levels and **K** LOX-1 evaluation by ELISA. Representation of **L** hsamiR-15b-5p, **M** hsa-miR-16-5p and N hsa-miR-195-5p levels measured by qRT-PCR. SIRT4 levels assessed by **O** ELISA and **P** immunoblotting. Mean ± SD, *n* = 3. M, molecular weight markers; lane 1, Ctr; lane 2 = LPS. *p < 0.05 vs. 0 µg/mL or Ctr; ^‡^*p* < 0.01 vs. 0 µg/mL or Ctr; ^¶^*p* < 0.001 vs. Ctr; n.s., non-significant. Statistical analysis of data was performed using Student’s *t*-test. **Figure S2.** LPS modulation of PCSK9 protein on HUVEC cells. **A** Representative intracellular PCSK9 protein content on TeloHAEC detected by FACS analysis. Detection of PCSK9 by **B** ELISA, **C** mRNA levels by qRT-PCR and **D** immunoblotting analysis on HUVEC. Mean ± SD, *n* = 3. M, molecular weight markers; lane 1, Ctr; lane 2, LPS. *p < 0.05 vs. Ctr; ^‡^*p* < 0.01 vs. Ctr. Statistical analysis of data was performed using Student’s *t*-test. **Figure S3.** *LPS-induced pyroptosis on HUVEC cells*. Representative FACS analysis of **A** pyroptosis, **B** intracellular NLRP3 levels, **C** lysosomes, **D** autophagy, **E** mitochondrial ROS levels and **F** annexin V-FITC and PI-staining on TeloHAEC. Q1: necrotic cells; Q2: late apoptotic cells; Q3: early apoptotic cells; Q4: viable cells. **G** Images and **H**, **I** cytometer analysis of pyroptosis on HUVEC. Mean ± SD, *n* = 3. Scale bars = 100 μm. ^‡^*p* < 0.01 vs. Ctr. Statistical analysis of data was performed using Student’s t-test. **Figure S4.** *Transfection with i-miR-15b*. TeloHAEC viability evaluated **A** after antagomir Negative Control (NC) and antagomiR hsa-miR-15b-5p (i-miR-15b) transfection and **B** after exposure to LPS on NC-transfected cells. Representative FACS analysis of **C** pyroptosis and **D** intracellular NLRP3 levels detected on TeloHAEC. Mean ± SD, *n* = 3. *p < 0.05 vs. NC; ^‡^*p* < 0.01 vs. NC; ^§^*p* < 0.001 vs. NC. Statistical analysis of data was performed using Student’s *t*-test. **Figure S5.** FACS analyses. Representative FACS analysis of **A** lysosomes, **B** autophagy, **C** mitochondrial ROS levels and **D** annexin V-FITC and PI-staining performed on TeloHAEC. Q1: necrotic cells; Q2: late apoptotic cells; Q3: early apoptotic cells; Q4: viable cells. **Figure S6.** *i-PCSK9 effects on LPS-induced pyroptosis and autophagy*. **A** TeloHAEC viability after treatment with LPS, i-PCSK9 or transfection with NC before i-PCSK9 and/or LPS stimulation. Representative images and FACS analysis of **B**, **C** pyroptosis and **D**, **E** autophagy performed on TeloHAEC. Mean ± SD, *n* = 3. Scale bars = 100 μm. ^¶^*p* < 0.001 vs. Ctr; ^§^*p* < 0.001 vs. NC; ^•^*p* < 0.05 vs. NC + LPS; ^&^*p* < 0.05 vs. LPS. Statistical analysis of data was performed using one-way ANOVAs.

## Data Availability

The datasets used and analysed during the current study are available from the corresponding author on reasonable request.

## Abbreviations

AFU: Arbitrary fluorescence units
AKT3: Protein kinase B3
Ang-2: Angiopoietin-2
ASC: Apoptosis-associated speck-like protein containing CARD
AU: Arbitrary units
DAPI: 4′,6-Diamidino-2-phenylindole
EC: Endothelial cells
eIF2a: Eukaryotic translation initiation factor 2A
FAK: Focal adhesion kinase
HUVEC: Human umbilical vein endothelial cells
i-miR-15b: Hsa-miR-15b-5p antagomiR
i-PCSK9: Proprotein convertase subtilisin-kexin type 9 inhibitor
ICAM1: Intercellular adhesion molecule 1
IGFBP-3: Insulin-like growth factor-binding protein-3
IL: Interleukin
LC3B II/I: Microtubule-associated proteins 1A/1B light chain 3B
LDH: Lactate dehydrogenase
LDL: Low-density lipoprotein
lncRNA: Long noncoding RNA
LOX-1: Lectin-like ox-LDL receptor-1
LPS: Lipopolysaccharide
MCP-1: Monocyte chemoattractant protein-1
miRNA: MicroRNA
mTOR: Mammalian target of rapamycin
NC: Negative control antagomiR
NF-κB: Nuclear factor kappa B
NLRP3: NLR family pyrin domain containing 3
NO: Nitric oxide
PCSK9: Proprotein convertase subtilisin-kexin type 9
qRT–PCR: Quantitative real-time PCR
ROS: Reactive oxygen species
SDS–PAGE: Sodium dodecyl sulfate–polyacrylamide gel electrophoresis
SIRT: Sirtuin
SOCS3: Suppressor of cytokine signaling 3
TNF-α: Tumor necrosis factor-α
TNIP2: TNF-α induced protein 3 (NFAIP3)-interacting protein 2
3′-UTR: 3′-Untranslated region
VCAM1: Vascular cell adhesion molecule 1
VEGF: Vascular endothelial growth factor
VEGFR2: Vascular endothelial growth factor (VEGF) receptor 2
