# Supplementary material for: MiR-15b-5p and PCSK9 inhibition reduces lipopolysaccharide-induced endothelial dysfunction by targeting SIRT4
Source: Cell Mol Biol Lett. 2023 Aug 16;28:66. doi: 10.1186/s11658-023-00482-5 (PMC10428548; doi:10.1186/s11658-023-00482-5)
Supplement: Supplementary file 1 — Additional file 1: Figure S1. LPS-mediated inflammation on HUVEC cells. A Cell viability on HUVEC exposed to LPS. Detection of B LDH, C NO, D VCAM1 E ICAM1, F MCP-1, G IL-1β and H IL-18 levels. Caspase-4 I activity and J levels and K LOX-1 evaluation by ELISA. Representation of L hsamiR-15b-5p, M hsa-miR-16-5p and N hsa-miR-195-5p levels measured by qRT-PCR. SIRT4 levels assessed by O ELISA and P immunoblotting. Mean ± SD, n = 3. M, molecular weight markers; lane 1, Ctr; lane 2 = LPS. *p < 0.05 vs. 0 µg/mL or Ctr; ‡p < 0.01 vs. 0 µg/mL or Ctr; ¶p < 0.001 vs. Ctr; n.s., non-significant. Statistical analysis of data was performed using Student’s t-test. Figure S2. LPS modulation of PCSK9 protein on HUVEC cells. A Representative intracellular PCSK9 protein content on TeloHAEC detected by FACS analysis. Detection of PCSK9 by B ELISA, C mRNA levels by qRT-PCR and D immunoblotting analysis on HUVEC. Mean ± SD, n = 3. M, molecular weight markers; lane 1, Ctr; lane 2, LPS. *p < 0.05 vs. Ctr; ‡p < 0.01 vs. Ctr. Statistical analysis of data was performed using Student’s t-test. Figure S3. LPS-induced pyroptosis on HUVEC cells. Representative FACS analysis of A pyroptosis, B intracellular NLRP3 levels, C lysosomes, D autophagy, E mitochondrial ROS levels and F annexin V-FITC and PI-staining on TeloHAEC. Q1: necrotic cells; Q2: late apoptotic cells; Q3: early apoptotic cells; Q4: viable cells. G Images and H, I cytometer analysis of pyroptosis on HUVEC. Mean ± SD, n = 3. Scale bars = 100 μm. ‡p < 0.01 vs. Ctr. Statistical analysis of data was performed using Student’s t-test. Figure S4. Transfection with i-miR-15b. TeloHAEC viability evaluated A after antagomir Negative Control (NC) and antagomiR hsa-miR-15b-5p (i-miR-15b) transfection and B after exposure to LPS on NC-transfected cells. Representative FACS analysis of C pyroptosis and D intracellular NLRP3 levels detected on TeloHAEC. Mean ± SD, n = 3. *p < 0.05 vs. NC; ‡p < 0.01 vs. NC; §p < 0.001 vs. NC. Statistical analysis [file 11658_2023_482_MOESM1_ESM.pdf]

MiR-15b-5p and PCSK9 inhibition reduces lipopolysaccharide-induced endothelial dysfunction by targeting SIRT4

Elisa Martino[1], Nunzia D'Onofrio[1]*, Anna Balestrieri[2], Luigi Mele[3], Celestino Sardu[4], Raffaele Marfella[4], Giuseppe Campanile[5], Maria Luisa Balestrieri[1]

[1]Department of Precision Medicine, University of Campania Luigi Vanvitelli, Via L. De Crecchio 7, 80138 Naples, Italy.

[2]Food Safety Department, Istituto Zooprofilattico Sperimentale del Mezzogiorno, Via Salute 2, 80055 Portici, Italy.

[3]Department of Experimental Medicine, University of Campania Luigi Vanvitelli, Via Luciano Armanni 5, 80138 Naples, Italy.

[4]Department of Advanced Clinical and Surgical Sciences, University of Campania Luigi Vanvitelli, Piazza Miraglia, 80138 Naples, Italy.

[5]Department of Veterinary Medicine and Animal Production, University of Naples Federico II, Via F. Delpino 1, 80137 Naples, Italy.

***Correspondence:** nunzia.donofrio@unicampania.it; Tel.: +39-081-5667519

Additional file 1

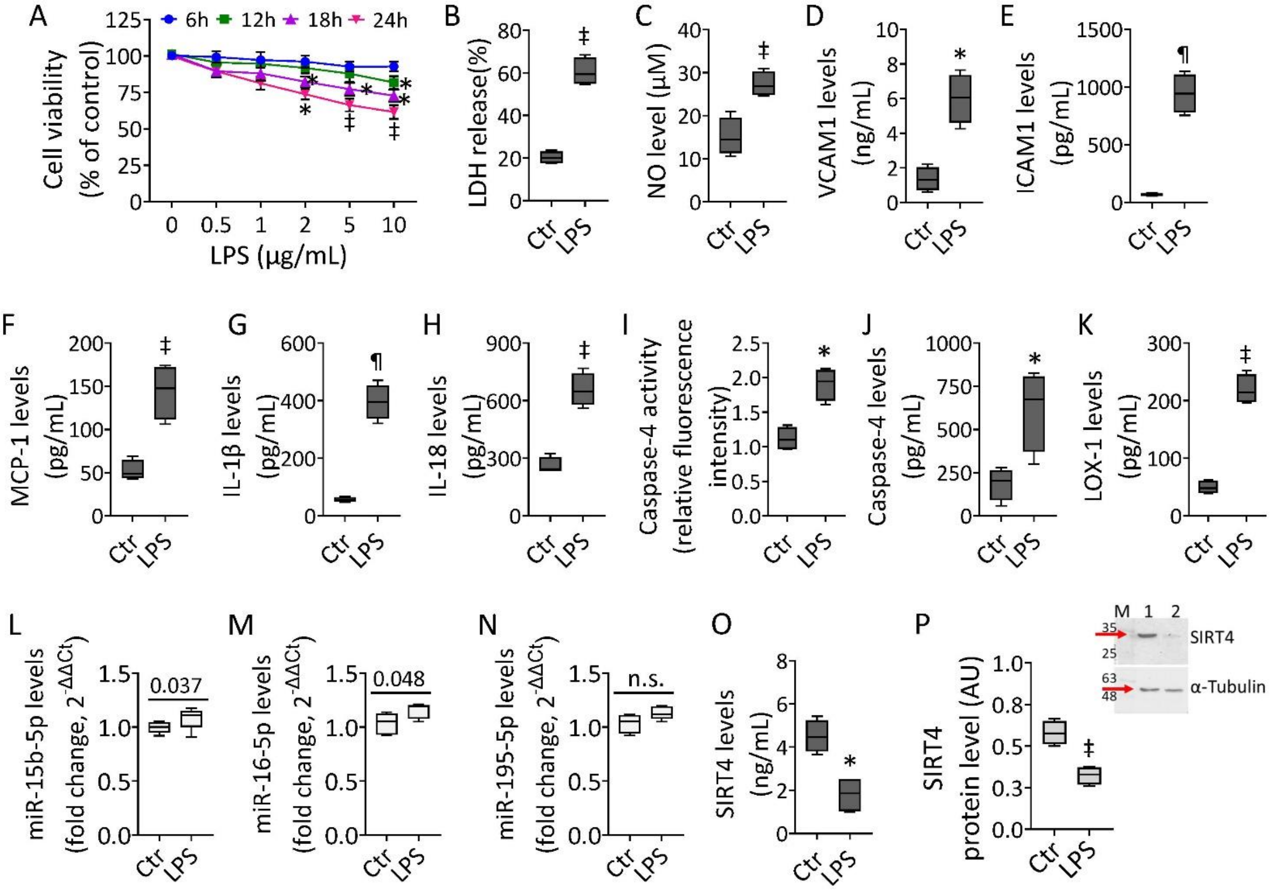

Fig. S1. *LPS-mediated inflammation on HUVEC cells*. (A) Cell viability on HUVEC exposed to LPS. Detection of (B) LDH, (C) NO, (D) VCAM1 (E) ICAM1, (F) MCP-1, (G) IL-1β and (H) IL-18 levels. Caspase-4 (I) activity and (J) levels and (K) LOX-1 evaluation by ELISA. Representation of (L) hsa-miR-15b-5p, (M) hsa-miR-16-5p and (N) hsa-miR-195-5p levels measured by qRT-PCR. SIRT4 levels assessed by (O) ELISA and (P) immunoblotting. Mean ± SD, n = 3. M = molecular weight markers; lane 1 = Ctr; lane 2 = LPS. *p<0.05 vs. 0 μg/mL or Ctr; ‡p<0.01 vs. 0 μg/mL or Ctr; ¶p<0.001 vs. Ctr; n.s., non-significant. Statistical analysis of data was performed using Student's t-test.

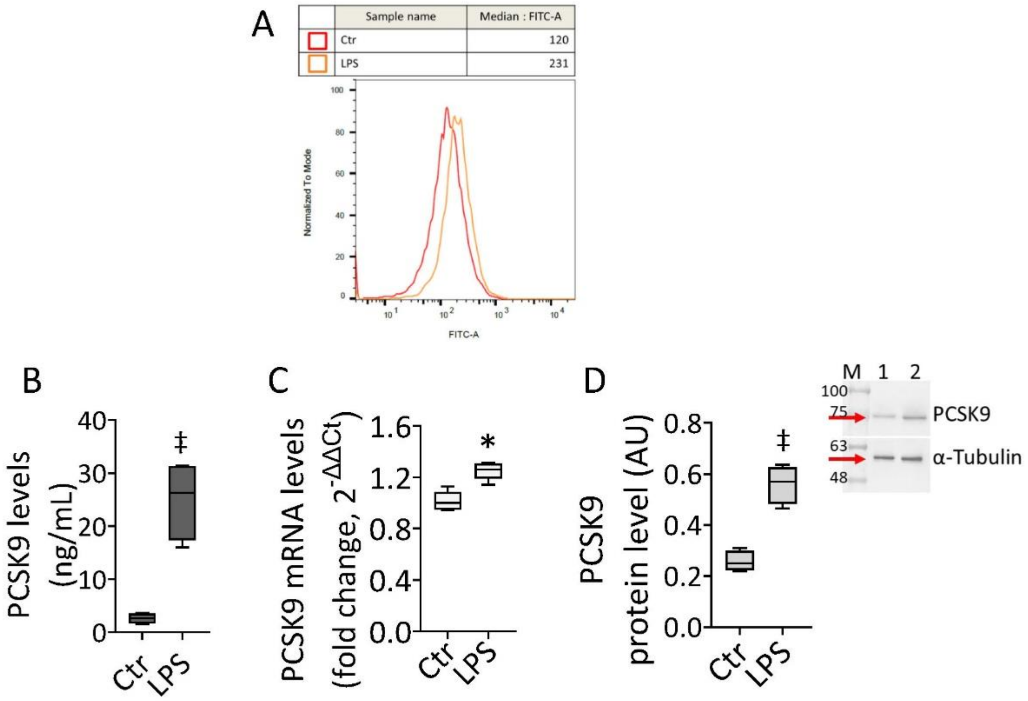

Fig. S2. *LPS modulation of PCSK9 protein on HUVEC cells*. (A) Representative intracellular PCSK9 protein content on TeloHAEC detected by FACS analysis. Detection of PCSK9 by (B) ELISA, (C) mRNA levels by qRT-PCR and (D) immunoblotting analysis on HUVEC. Mean ± SD, n = 3. M = molecular weight markers; lane 1 = Ctr; lane 2 = LPS. *$p<0.05$ vs. Ctr; ‡$p<0.01$ vs. Ctr. Statistical analysis of data was performed using Student's t-test.

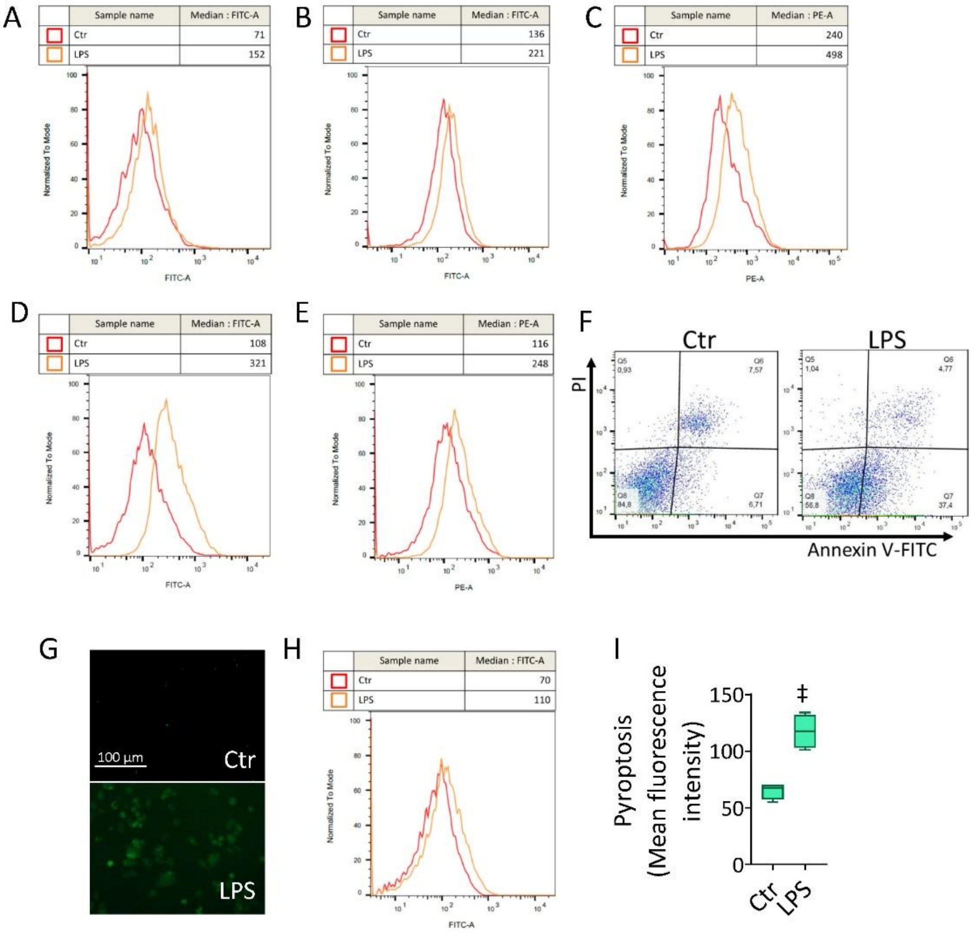

Fig. S3. *LPS-induced pyroptosis on HUVEC cells*. Representative FACS analysis of (A) pyroptosis, (B) intracellular NLRP3 levels, (C) lysosomes, (D) autophagy, (E) mitochondrial ROS levels and (F) annexin V-FITC and PI-staining on TeloHAEC. Q1: necrotic cells; Q2: late apoptotic cells; Q3: early apoptotic cells; Q4: viable cells. (G) Images and (H,I) cytometer analysis of pyroptosis on HUVEC. Mean ± SD, n = 3. Scale bars = 100 µm. ‡$p<0.01$ vs. Ctr. Statistical analysis of data was performed using Student’s t-test.

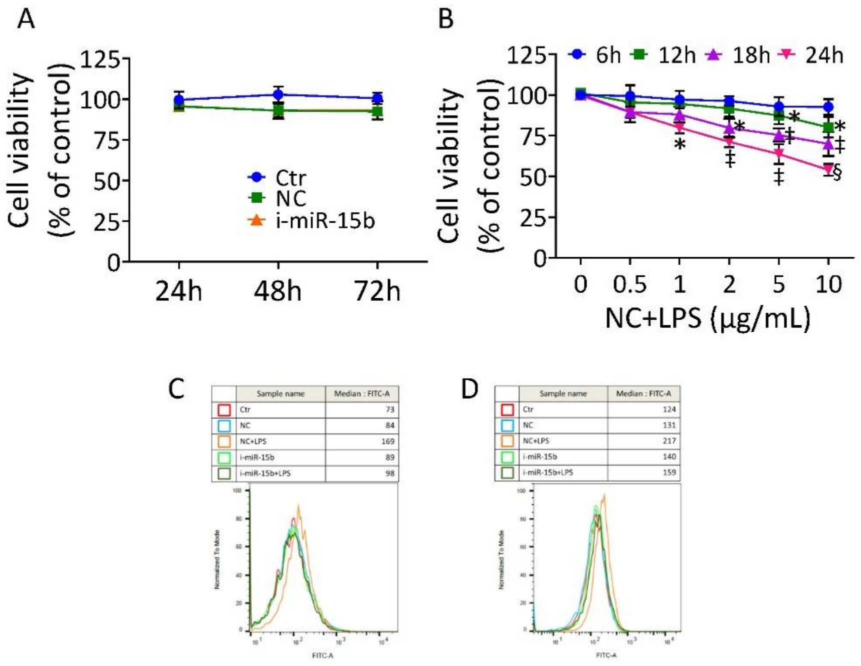

Fig. S4. *Transfection with i-miR-15b.* TeloHAEC viability evaluated (A) after antagomir Negative Control (NC) and antagomiR hsa-miR-15b-5p (i-miR-15b) transfection and (B) after exposure to LPS on NC-transfected cells. Representative FACS analysis of (C) pyroptosis and (D) intracellular NLRP3 levels detected on TeloHAEC. Mean ± SD, n = 3. *p<0.05 vs. NC; ‡p<0.01 vs. NC; §p<0.001 vs. NC. Statistical analysis of data was performed using Student's t-test.

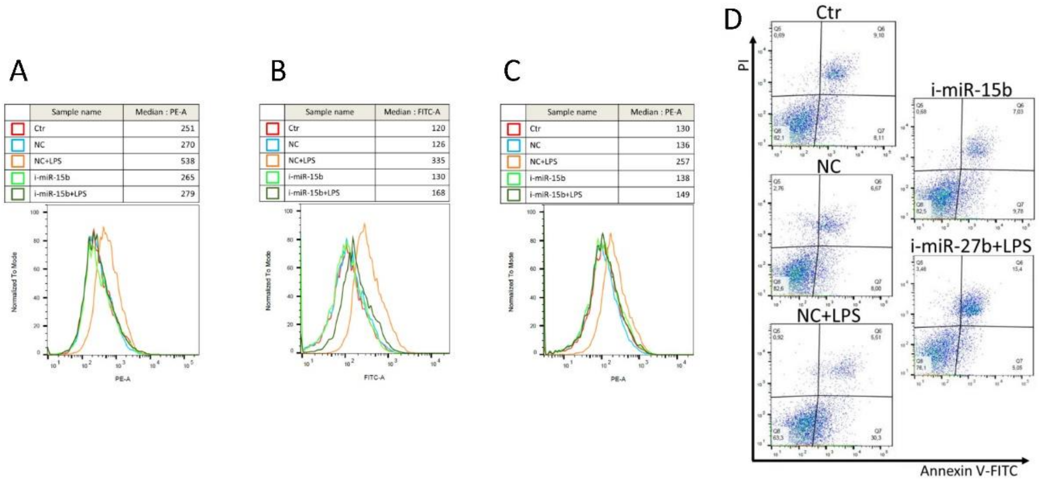

Fig. S5. *FACS analyses.* Representative FACS analysis of (A) lysosomes, (B) autophagy, (C) mitochondrial ROS levels and (D) annexin V-FITC and PI-staining performed on TeloHAEC. Q1: necrotic cells; Q2: late apoptotic cells; Q3: early apoptotic cells; Q4: viable cells.

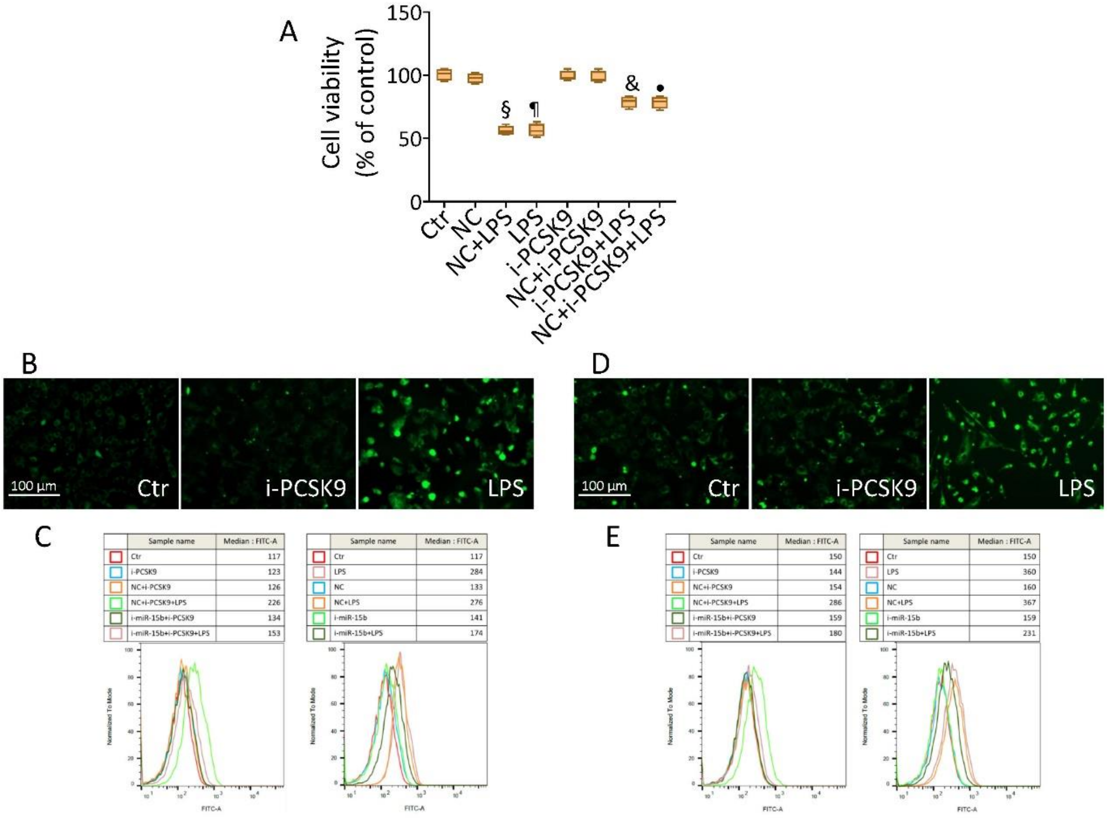

Fig. S6. *i-PCSK9 effects on LPS-induced pyroptosis and autophagy*. (A) TeloHAEC viability after treatment with LPS, i-PCSK9 or transfection with NC before i-PCSK9 and/or LPS stimulation. Representative images and FACS analysis of (B,C) pyroptosis and (D,E) autophagy performed on TeloHAEC. Mean ± SD, n = 3. Scale bars = 100 μm. ¶$p<0.001$ vs. Ctr; §$p<0.001$ vs. NC; •$p<0.05$ vs. NC+LPS; &$p<0.05$ vs. LPS. Statistical analysis of data was performed using one-way ANOVA.
